# Relevance and Safe Utilization of Amino Acids in Supplements for Human Nutrition: Lessons from Clinical and Preclinical Studies

**DOI:** 10.3390/nu18020296

**Published:** 2026-01-17

**Authors:** François Blachier

**Affiliations:** Unité Mixte de Recherche Physiologie de la Nutrition et du Comportement Alimentaire, Université Paris-Saclay, AgroParisTech, INRAe, 91120 Palaiseau, France; francoismichel.blachier@gmail.com

**Keywords:** amino acid supplementation, amino acid metabolism, safety of use, no-observed-adverse-effect level, amino acid requirement, targeted subpopulations

## Abstract

Amino acid availability is central for the synthesis of macromolecules and numerous bioactive compounds. Amino acids are also involved in ATP production, cell signaling, and the epigenetic regulation of gene expression in human cells. From clinical and experimental studies, it appears that supplementation with specific amino acids may be relevant to correct for amino acid deficiency in the case of insufficient supply from dietary proteins with regards to the amounts needed for optimal metabolism and physiological functions. Clinical and experimental arguments suggest that amino acid supplementation may be indicated in specific situations under a specific nutritional context. However, it is essential not to overdose with excessive quantities of amino acids in supplements beyond the upper levels of safe intake (ULSI). In this narrative review, I recapitulate the protein and amino acid requirements for the general population and for subgroups of the population, and these requirements are compared to the usual consumption. Typical examples of clinical trials showing the benefits from amino acid supplementation in different physiological and pathophysiological contexts are presented together with results obtained from experimental studies. Parameters such as the no-observed-adverse-effect-level (NOAEL) values used to determine the ULSI for amino acid supplementation are defined, and values determined in clinical trials are given and discussed. Finally, prospects for future research in the field are proposed.

## 1. Introduction

The 20 common amino acids (and the uncommon amino acid selenocysteine) are well known to represent the building blocks for protein synthesis in the different tissues and organs of the human body. In addition, specific amino acids such as glycine, aspartate, and glutamine are precursors for the synthesis of the purine and pyrimidine rings of RNAs and DNA in cells. These amino acids are also used in different human cell phenotypes for the synthesis of the free energy donor adenosine triphosphate (ATP) [1].

Amino acids are precursors for the synthesis of numerous bioactive peptides [2] as well as for the synthesis of metabolic regulators. *N*-acetylglutamate for instance is synthesized from the amino acid glutamate, and this compound is acting as an allosteric cofactor for carbamylphosphate synthetase I, an enzyme involved in the liver urea cycle [3]. Several neurotransmitters (such as serotonin, dopamine, adrenaline, noradrenaline, and gamma aminobutyrate), small size hormones like melatonin, and pigments like melanin require specific amino acids as precursors for their synthesis. In addition, hormonal secretion can be stimulated by several specific amino acids [1]. Some amino acids, mainly alanine and glutamine, are precursors for the synthesis of glucose [4,5] and serine/methionine are involved in the synthesis of phospholipids. Specific amino acids can be used as oxidative substrates in different cell phenotypes within tissues and organs allowing mitochondrial ATP synthesis.

Amino acids are also used as precursors for numerous bioactive compounds with central metabolic and physiological functions. Several among the 20 common amino acids are involved in cell signaling in target tissues. The branched-chain amino acids leucine, isoleucine, and valine for instance are involved through specific signaling pathways in the regulation of protein, glucose, and lipid metabolism [6]. Some amino acids are precursors of compounds involved in central physiological functions. Arginine for instance is the precursor of nitric oxide (NO) which is involved notably in vascular muscle relaxation [7]. Some amino acids are required for nitrogenous waste disposal. Regarding this last point, the functioning of the urea cycle, for example, involves amino acids such as arginine and aspartate. Finally, an amino acid such as methionine is involved, through its conversion into S-adenosylmethionine, in DNA and histone methylation [8]. These methylation processes are an important component for the epigenetic regulation of gene expression in human cells [9].

**Figure 1:**
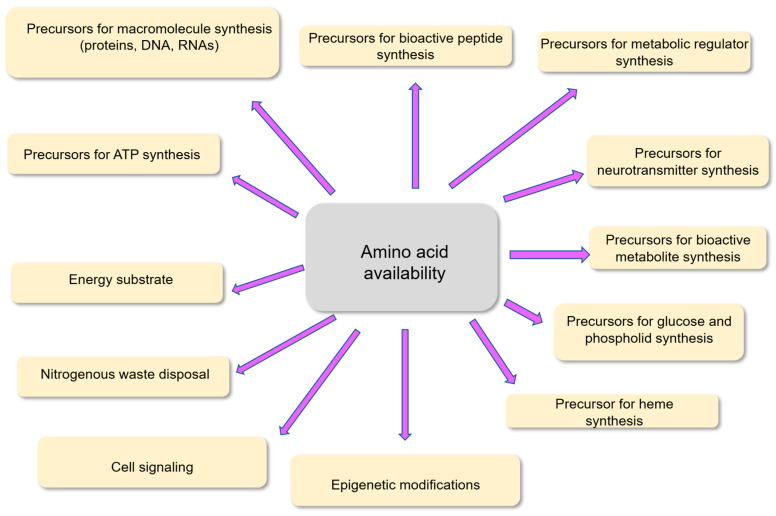
Schematic representation of the implication of amino acids as precursors for numerous compounds, as energy substrates, and as modulators of cell signaling. The compounds produced from amino acids include macromolecules and smaller molecules such as bioactive peptides, neurotransmitters, bioactive metabolites, ATP, glucose, and phospholipids. Specific amino acids are used as energy substrates in different cells within tissues. Some amino acids are involved in different physiological functions through cell signaling, epigenetic modifications, and utilization in metabolic pathways involved in nitrogenous waste disposal.

The percentages of amino acids involved as precursors for the synthesis of the different amino acid-derived compounds are evidently much different according to the pathways of use considered. As we will see below, protein renewal in the body requires large amounts of amino acids. In contrast, as a rule of thumb, the utilization of amino acids for compounds such as metabolic regulators, gaseous mediators (like nitric oxide and hydrogen sulfide), neurotransmitters, and tripeptides (like glutathione) represents only a minor part of their total utilization in the body. However, as observed in animal models, in specific situations such as infection and intestinal inflammation, glutathione synthesis from the three amino acid precursors cysteine, glutamate, and glycine is markedly increased. In such situations, the utilization of cysteine for the synthesis of glutathione may represent a significant part of the whole-body utilization of this amino acid [10,11]. The main roles of amino acids in the human body are briefly recapitulated in Figure 1.

**Figure 2:**
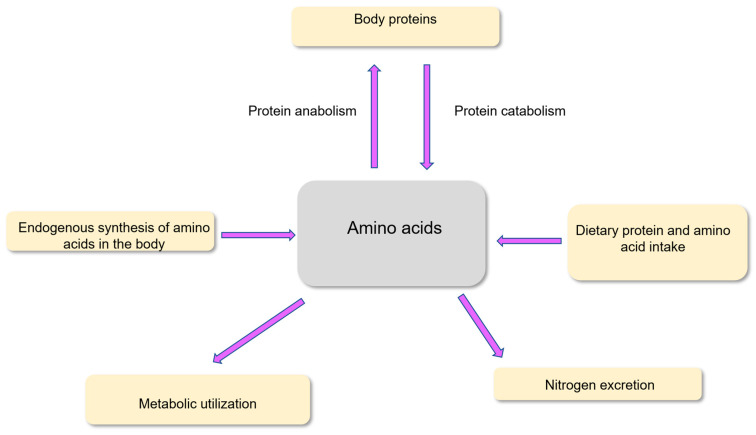
Schematic representation of the supply and utilization of amino acids in the human body. Amino acids originate from diet, from the endogenous synthesis from various precursors, and from body protein catabolism. Amino acids are used for metabolic utilization in numerous pathways including notably body protein synthesis.

Adequate amounts of amino acids, mostly from dietary proteins and to a minor extent from amino acids in free form in the diet, must be regularly provided. Also, amino acids are provided from endogenous synthesis and protein recycling in the body. Amino acid availability from these different sources must meet the metabolic and physiological needs of the different tissues. Amino acid utilization is also required for the excretion of nitrogenous compounds within the biological fluids (urine, feces, etc.) (Figure 2). A large part of the utilization of amino acids is devoted to the renewal of the body proteins. Indeed, in healthy adults weighing 70 kg, approximately 300–400 g body proteins are renewed every day among the 10–12 kg mass of body proteins [12].

Among the 20 common amino acids present in dietary proteins, 9 of them (isoleucine, leucine, valine, methionine, tryptophan, threonine, phenylalanine, histidine, and lysine) must be provided by the diet because the body is not able to synthesize them (or not in sufficient quantities) to meet the body’s requirement [13]. These amino acids are then considered as indispensable. However, they are not all equivalent since for some of them, like lysine and threonine, no metabolic capacities for their synthesis have been identified [14], while for some others, minor capacities of synthesis can be detected in some tissues. For instance, homocysteine, which is found in low amounts in some dietary products [15], can represent a precursor for the synthesis of methionine through vitamin B12-dependent pathways [16]. Different criteria are used to estimate the quality of dietary proteins originating either from animal and plant sources or from microorganisms, and these criteria include the digestibility of the alimentary proteins as well as their content in indispensable amino acids [17].

The other 11 amino acids (thus alanine, arginine, asparagine, aspartate, cysteine, glutamate, glutamine, glycine, proline, serine, and tyrosine) by difference are considered as not indispensable (or dispensable), and for these ones, anabolic pathways from other amino acids or non-amino acid precursors have been characterized and found to allow the significant endogenous production of these amino acids [1].

Among these 11 amino acids, another subclassification has been made gathering 6 amino acids considered as conditionally indispensable. These amino acids, although being synthesized in significant amounts within the body, are not sufficiently produced in different physiological and pathophysiological situations to cover the metabolic and physiological needs [18]. These conditionally indispensable amino acids are arginine, cysteine, glutamine, glycine, proline, and tyrosine. Regarding arginine, this amino acid becomes for instance indispensable in severely burned patients [19], while cysteine supplementation appears clinically relevant in subgroups of critically ill patients. In these patients, such supplementation can increase the endogenous synthesis of the cysteine-derived tripeptide glutathione [20]. Glutathione is well known to limit the severity of chronic inflammation in different clinical contexts [20,21,22]. Regarding glutamine, this amino acid has been shown to become indispensable in patients undergoing severe catabolic states like the one encountered in sepsis [23] while glycine may become indispensable in the late stages of pregnancy [24].

In order to evaluate the relevance of supplementation with one or several amino acids in a given physiological/pathophysiological situation, it is important to cross various parameters including protein and amino acid metabolism, protein and amino acid requirement, usual consumption, and clinical endpoints determined in clinical intervention studies, together with the results of experimental studies which can provide a better understanding of the mechanisms at the basis of the observations made.

The search strategy and the literature selection criteria for the present narrative review were based on the publications available on PubMed using several keywords used individually and in combination with no restriction on the covered time frame. These keywords are the names of the 20 common proteinogenic amino acids, ornithine, citrulline, amino acid supplementation, amino acid metabolism, human metabolism, amino acid requirements, amino acid usual consumption, safety of use, no-observed-adverse-effect level, upper level of safe intake, elderly, athletes, sarcopenia, intestinal mucosal healing, inflammatory bowel diseases, chronic kidney diseases, metabolic syndrome, weight loss programs, osteoporosis, and cognitive decline.

In this narrative review, I present different situations where supplementation with one specific or with mixtures of different amino acids showed beneficial effects in clinical trials and/or in experimental studies. I then present what is known from clinical studies about the upper limits for the safe utilization of amino acids in supplements.

## 2. Protein and Amino Acid Requirement Along the Life Cycle and Comparison with Usual Consumption in Different Geographical Areas

The dietary protein requirement in adult humans can be defined basically as the amounts of dietary proteins that will maintain the body protein mass in individuals with moderate levels of physical activity. In children and pregnant or breast-feeding women, the amounts of dietary proteins that will ensure the maintenance of body protein mass must consider the additional amounts of proteins that will ensure adequate tissue deposition in children and pregnant women, or the secretion of milk with an adequate composition in lactating women, respectively. From the notion of protein requirement, the recommended protein nutritional intake corresponds to the value which covers the requirements of most of the population, on a statistical basis of 97.5% of the individuals among the populations studied. This value is generally close to the average requirements to which two standard deviations are added [25]. Incidentally, it is just fair to recognize that only a minor proportion of the individuals among the general population can estimate, even roughly, their own alimentary protein consumption.

### 2.1. Protein Requirement in Different Situations

The average requirement for proteins in the general population of healthy adults has been estimated to be 0.65 g proteins per kg body weight per day, while the recommended dietary intake is 0.83 g proteins per kg body weight per day, thus representing 58.1 g of good quality dietary proteins for an individual weighing 70 kg [26]. However, it is important to emphasize immediately that this recommendation does not apply to certain subgroups of the population. Notably, in individuals with chronic kidney diseases, the recommended dietary protein intake is lower, being between 0.60 and 0.80 g per kg body weight per day [27,28]. Indeed, higher protein intake may lead to increased intraglomerular pressure and glomerular hyperfiltration, thus causing damage to the glomerular structure. On the other hand, a recent article reports on the association of higher protein intake with lower mortality in older adults with moderate chronic kidney disease [29]. This indicates a careful choice to set the optimal protein intake depending on the clinical status of the patients with chronic kidney disease and other comorbidities (such as sarcopenia, as will be discussed in the next parts of this review).

These values can be compared with the usual consumption (UC) in different geographical areas. In Europe and the USA, the average dietary protein consumption is approximately 85 g per day in adults [30,31], and thus largely above the recommended intake. However, this average value must not mask subgroups of the population for whom UC can be much lower. To take an example, in vegans, the total protein intake is lower when compared to the intake in non-vegan groups [32] and approximately one third of vegans are found below the adequate protein intake [33].

Some other countries are below the average protein consumption found in the Western world. In Thailand for instance, the average consumption of proteins is 63.4 g per day [34], and thus close to the recommended intake. In some countries, the UC is largely below the recommended intake. For instance, in Southern Ethiopia, the prevalence of inadequate dietary protein intake is found in 94% of the female population [35]. More than 700 million persons in the human population do not meet the energy and nutritional requirements (including protein requirement) (UNICEF 2024 report).

In infants between birth and 3 years old, the estimation of the protein requirement is within 0.76–1.80 g proteins/kg/day, with a recommended intake between 0.94 and 2.60 g proteins/kg/day [36]. In children between 4 and 10 years, the protein requirement is 0.70 g proteins/kg/day, and the recommended intake is 0.88 g proteins/kg/day. In adolescents (11–18 years), the protein requirement and the recommended intake is between 0.63 and 0.72 g proteins/kg/day, and between 0.78 and 0.90 g proteins/kg/day, respectively. These estimations have also been made in pregnant women, averaging between 0.65 and 0.73 g proteins/kg/day and between 0.82 and 1.00 g proteins/kg/day for the requirement and the recommended intake, respectively. In breast-feeding women, the average requirement and the recommended intake are 0.8 and 1.1 proteins/kg/day, respectively [36]. However, it is important to mention here that protein requirements in these human subgroups are originating from only a limited number of studies [37]. Indeed, the range of average protein requirements in infants appears particularly large due to differences between the estimates.

The requirement and recommendation for the elderly are generally higher than for young adults, and in several recent publications, the recommended intake has been evaluated to range between 1.0 and 1.6 g proteins/kg/day, thus representing between 70 and 105 g of good quality proteins [38,39,40,41]. The average protein requirement along the life cycle in heathy individuals is recapitulated in Table 1.

Despite the general recommendation to increase the intake of dietary proteins in the elderly population, protein–energy malnutrition is detected in approximately 1% of this population in Northern Europe, 11% in Southern Europe, 15% in Central and West Africa, and 18% in Australia/New Zealand, while protein–energy malnutrition represents as much as 25% of the elderly population in South-East Asia [42].

In specific situations such as athletes practising endurance or strength sports, the recommended intake of dietary proteins is higher than the one in individuals with moderate physical activity, being situated between 1.3 and 1.8 g proteins/kg/day (91–126 g proteins per day for individuals weighing 70 kg) [43]. In athletes practising resistance exercise, the proposed recommendation for total protein uptake (dietary protein and protein in supplement) is even higher representing as much as 2.0 g/kg/day (140 g protein per day for 70 kg body weight) [44].

### 2.2. Indispensable Amino Acid Requirement in Adults and Comparison with Usual Consumption in Western Countries

The requirement and usual consumption (UC) of indispensable amino acids have been described in several reports [36,45,46] and in one recent review [47]. Consumption data for indispensable amino acids in food and supplements are expressed in mg per kg body weight in Table 2 to allow comparison with the requirements. As indicated in this table, the requirements for indispensable amino acids in adults are somewhat different for some amino acids when comparing the values given in the three reports. However, it is worth noting that for methionine and phenylalanine, the presented requirement values do not relate exactly to the same parameters.

The Food and Nutrition Board and Institute of Medicine (FNB/IOM) data for the usual consumption (UC) referred to the USA and Canadian population for all life stages and gender groups [44]. In the same table are presented the UC of indispensable amino acids by European adults from the EPIC cohort (Nutr. Metab. Cardiovasc. Dis. 2022, NMCD values) [48,49]. These mean values of UC are similar when comparing the two populations, being higher than the requirement for all the nine indispensable amino acids.

## 3. Amino Acid Supplementation in Specific Situations: Lessons from Clinical Trials and Experimental Studies

The beneficial effects of amino acid supplementation have been tested in physiological and pathophysiological situations of presumed insufficient dietary protein intake with regards to the requirements. This may be for instance the case in the elderly population, who are recognized as at risk for sarcopenia, and thus who may experience progressive loss of muscle mass and strength [50].

### 3.1. Leucine Supplementation in the Sarcopenic Elderly

The rationale for testing supplementation with specific amino acids together with protein of good quality in sarcopenic elderly people is rather robust for several reasons. Firstly, in a context of the increased requirement for proteins in the elderly, muscle protein synthesis is reduced in aging adults [51], and such a reduction is paralleled by a reduction in protein synthesis within human muscle mitochondria [52]. Such a reduction occurs in conjunction with a reduced mitochondrial capacity for ATP production [53]. Secondly, protein synthesis in the muscles of elderly people is less responsive to amino acids than in young subjects, a situation defined as part of anabolic resistance [54]. The positive effects of higher versus lower levels of proteins in foods on the preservation of lean mass have been observed in older adults [55,56].

The branched-chain amino acids (BCAAs) leucine, isoleucine, and valine have raised considerable interest because of their different metabolic and physiological functions. BCAAs are used for protein synthesis in mammalian cells, and transaminated in the presence of α-ketoglutarate, allowing the production of glutamate and the corresponding α-ketoacids [57,58]. Each α-ketoacid can then undergo several steps of conversion resulting in the synthesis of acetyl-CoA and succinyl-CoA. These compounds can then enter the tricarboxylic acid cycle allowing the synthesis of reduced equivalents. These equivalents are finally used in the mitochondrial respiratory chain allowing ATP synthesis. BCAAs are precursors not only for the dispensable amino acid glutamate, but also for alanine and glutamine. BCAAs are involved in the regulation of metabolic pathways engaged in glucose and lipid metabolism, and in protein synthesis in skeletal muscles. Regarding more specifically leucine, this BCAA has been shown to act on muscle protein synthesis through the stimulation of the mTORC1/P70S6K signaling pathway, which represents the major myocellular signaling pathway [59].

Leucine (52 mg/kg body weight) has been found to be able to increase protein synthesis in skeletal muscles when measured over the 5 h period of feeding [60]. As expected, leucine supplementation is paralleled by the increased circulating concentration of this amino acid. Generally, large increases in peripheral indispensable amino acid concentrations (notably leucine) are necessary to drive significant increases in muscle and whole-body protein synthesis [61]. Then, leucine has been tested for its effects on muscle mass and strength in the elderly in controlled clinical trials with and without physical exercise. The results of these trials are rather heterogeneous due to different volunteer characteristics and experimental designs (doses of leucine given orally and duration of supplementation, quantity and quality of proteins given together with the amino acid supplements, presence of other amino acids and vitamins in the supplements, chosen endpoints, etc.).

In the double blind, randomized controlled clinical study by Rondanelli and collaborators, supplements containing 10.9 g indispensable amino acids including 4 g of leucine together with 22 g of whey protein and vitamin D, when associated with physical activity for 12 weeks, increased skeletal muscle mass and handgrip strength in sarcopenic elderly subjects [62]. In other randomized controlled studies, supplements containing 2.8 g leucine, 20 g whey protein, and vitamin D given for 4–8 weeks increased muscle mass and improved physical performance in older adults [63]. Supplements containing 3 g leucine, 20 g whey proteins, and vitamin D, when given for 13 weeks to volunteers, increased muscle mass in older adults [64]. However, in these trials, the respective roles of leucine, of the amino acids originating from whey proteins, and of vitamin D could not be delineated.

From these studies, several comments can be made. Firstly, considering that 1 g whey protein brings 81 mg leucine [65], the total amount of leucine brought by the whey protein and supplements in these three studies are between 4 and 6 g. We can compare these values with the approximative amount of leucine that is contained within 91 g of proteins, and thus the mean recommended intake for old adults weighing 70 kg. By considering that the usual consumption of leucine in North America is 5.8 g/day from a diet containing 85 g proteins, 91 g proteins provide 6.2 g leucine, a value close to the upper level of leucine provided in the three clinical trials mentioned above. In other words, the supply of 6 g leucine from good quality dietary proteins and from supplements (together with an adequate supply of vitamin D) should be adequate in providing the adequate supply of leucine and of other amino acids in older adults in terms of skeletal muscle metabolism and physiology. Indeed, it must be kept in mind that optimal protein synthesis in muscles requires a sufficient supply of all amino acids, the ones that are provided by whey proteins and amino acids in their free form in supplements. Secondly, the requirement for leucine appears higher in older than young adults [66]. This suggests that in seniors, leucine could represent a limiting amino acid for protein synthesis, for utilization in pathways allowing ATP production, and even maybe for signal functions in skeletal muscles when the supply of protein is below the recommendation for older adults.

It is of major importance to consider both the age-related decline in appetite observed in subpopulations among the elderly and the satiating properties of proteins [67,68,69]. Studies in older adults suggest that the supplements with indispensable amino acids are non-satiating (or at least not as satiating as a high protein diet) in both acute and medium-term experiments [70,71,72,73]. Then, supplements with indispensable amino acids with an abundant leucine content represent a valuable strategy to enhance the anabolic properties of a meal containing a suboptimal supply of proteins in older adults with little appetite.

The exhaustive mention of all the randomized controlled studies dealing with leucine supplementation in the elderly in different experimental contexts is outside the scope of this review, and readers are referred to excellent reviews on that topic [74,75,76,77,78,79]. From these reviews, several important conclusions can be drawn. Leucine supplementation exerts beneficial effects on the muscle protein fractional synthetic rate, muscle mass, and lean body mass in older adults prone to sarcopenia or with diagnosed sarcopenia. Regular physical exercise together with supplements with indispensable amino acids (with high leucine content) and/or whey proteins together with vitamin D improve the maintenance or gains of skeletal muscle mass and total lean mass in sarcopenic older adults. Importantly, the effects of physical exercise and dietary supplements appear synergistic. Since concentrations of some amino acids, including not only BCAAs but also dispensable amino acids such as arginine, glutamine, glycine, and serine, are diminished in the plasma of older adults, further clinical trials are needed to test supplements with combinations of individual amino acids (and some of their metabolites) for their effects in limiting sarcopenia in older adults prone to sarcopenia.

### 3.2. Amino Acid Supplementation in Athletes

Branched-chain amino acids (BCAAs) are among the most popular supplements, marketed under the promise that they enhance muscular adaptations to physical exercise. Despite their prevalent consumption among athletes and general sportsmen and sportswomen, large controversies remain in this sports nutrition field. Although it appears clearly that, in the context of sufficient protein–energy intake, supplementation with BCAAs has little or no efficacy for enhancing the gains of muscle mass and strength-based physical performance during resistance training, there is some clinical evidence that muscle loss may be minimized by BCAAs in cases of protein–energy restriction [80,81].

Among BCAAs, leucine given by intravenous infusion in healthy volunteers has been shown to decrease protein degradation [82]. BCAA supplementation (100 mg BCAAs/kg body weight) increases protein synthesis in muscles during the recovery phase (2 h) after 1 h of endurance sports [83]. In volunteers, BCAA infusion increases the phosphorylation of proteins involved in the translation of mRNA corresponding to proteins involved in the synthesis of muscle proteins [84]. In a clinical trial, oral supplementation with arginine and the nine indispensable amino acids (including leucine which represents 20% in the mass of the amino acid mixture (40 g)) for 3 h after one-hour heavy resistance training results in the transition from net muscle protein degradation to net protein synthesis when compared to the placebo [85].

Experimental studies in animal models have revealed the mechanisms involved in the biological effects of BCAAs on skeletal muscles. Notably, in the piglet model, leucine has been shown to upregulate the mammalian target of rapamycin (mTOR) complex (mTORC)1, an element of the signaling cascade involved in muscle protein synthesis [59,86]. In the rat model, it has been demonstrated that physical exercise promoted BCAA oxidation in skeletal muscles [87]. The mechanisms at the basis of such effects involve the activation of the branched-chain α-ketoacid dehydrogenase (BCKDH) complex which catalyzes the second step reaction in the BCAA catabolic pathway [88].

The role of creatine for the metabolism and physiology of skeletal muscles in physical exercise is out of the scope of the present review because this compound does not belonging to the 21 amino acids used for protein synthesis, but is synthesized from arginine, glycine, and methionine [89]. The interested readers are referred to recent reviews on that topic [90,91].

### 3.3. Amino Acid Supplementation for Intestinal Mucosa Healing

Inflammatory bowel diseases (IBDs) are characterized by mucosal lesions in the intestine which are associated with exacerbated immune functions of still unclear etiology [92]. IBD, mainly Crohn’s disease (CD) and ulcerative colitis (UCol), are identified by the chronic inflammation of the mucosa with alternating relapse and remission periods. In CD, remission episodes offer the possibility of mucosal healing, allowing in some cases the total disappearance of all mucosal ulcerations [93]. However, in clinical practice, this endpoint is not easy to achieve. For patients with UCol, mucosal healing is described as the absence of friability, blood, erosions, and ulcers in all segments of the gut mucosa [94,95]. An advanced mucosal healing is associated with sustained remission, improved clinical outcomes, and reduced rates of hospitalization, thus explaining why mucosal healing represents a therapeutic goal for gastroenterologists in charge of CD and UCol patients [96,97,98,99].

Some clinical and experimental studies report on the requirement of dietary proteins in the process of intestinal mucosa healing and on the implications of specific amino acids used in supplements for accelerating this process [100]. Mucosal healing is a combination of different protein–energy dependent processes which allow the restoration of the continuity of the intestinal epithelium [98,101]. Indeed, poor nutritional status may characterize patients with IBD in the active phase because of anorexia, intestinal malabsorption, increased intestinal losses, and increased catabolism [102]. Poor nutritional status is likely to affect the efficiency of mucosal healing, and protein–energy malnutrition together with deficiencies in minerals and vitamins have been reported in patients with IBD [103,104,105]. Overall, elemental formulas with amino acids do not appear to be more efficient for clinical remission in adults and children than polymeric formulas containing whole proteins [106,107,108,109].

Although the protein requirements for intestinal mucosal healing in CD and UCol patients in remission have not been determined, there are some reasons, mostly from experimental studies, to presume that the daily protein requirements in IBD patients in remission may differ from those in healthy adults. The European Society for Clinical Nutrition and Metabolism recommends increasing the protein intake to 1.2–1.5 g per kg body weight per day in adult patients with active IBD, and to maintain an intake of 1 g proteins per kg body weight per day during remission [110].

In the mouse model, colitis resolution with mucosal healing, when measured from 2 to 23 days after colitis induction with dextran sodium sulfate, is paralleled by an increased fractional protein synthesis rate in the colon when compared with the situation observed in the animals before colitis induction [111]. Some experimental studies point to the potentially beneficial effects of moderately increased dietary protein ingestion and of supplementation with specific amino acids for mucosal healing after an inflammatory episode. In a mouse model of chemically induced colitis, a moderately high protein diet (30% of energy supplied by casein and whey proteins) given to animals at different times after the time of maximal colitis intensity (up to 21 days after dietary intervention) has been tested. This diet shows beneficial effects during the epithelial repair process within the colonic mucosa when compared with animals receiving a normoproteic diet (14% of energy as proteins) [112]. In sharp contrast, a frankly high protein diet (53% of energy supplied by proteins) resulted in the worsening of the inflammation both in intensity and duration. Although the reasons that would explain the deleterious effects of a high protein diet on the colonic mucosa of mice in the situation of experimental colitis remains unknown, high protein diet consumption has been shown to be associated with changes in the amounts of bacterial metabolites within the colonic luminal fluid in a way that is globally deleterious for the colonic epithelium [113]. Although a high protein diet given for 3 weeks to healthy subjects does not result in any sign of mucosal inflammation in the large intestine [114], such a diet may be deleterious for patients with inflammatory bowel disease in remission. Indeed, following one-year patients with ulcerative colitis in remission, it was found that patients with the highest dietary consumption of protein and sulfate experienced a threefold increase for the risk of relapse when compared to patients with the lower protein intake [115]. A plausible explanation for such results could be related to the excessive production of amino acid-derived bacterial metabolites, and notably of hydrogen sulfide. Hydrogen sulfide, which is synthesized from sulfate and cysteine by the intestinal microbiota, severely inhibits mitochondrial energy metabolism in colonic epithelial cells when present in excessive concentration, and a high protein diet increases the luminal and fecal sulfide concentrations [116]. Overall, these data suggest that a moderate increase in dietary protein intake facilitates mucosal healing in the colon, but that excessive intake is deleterious. This indicates a tricky choice to set the correct level of proteins to bring during remission. In such a context, supplementation with specific amino acids instead of simply increasing the dietary protein intake may appear valuable.

Supplementation with threonine, serine, proline, and cysteine (15, 10, 15, and 7.2 g/kg diet, respectively) have been shown to increase mucin synthesis when given 8 days before experimental colitis induction in the rat model and 28 days thereafter [117]. Since an intact mucus layer is necessary for providing an “initial seal” after intestinal mucosa injury [118,119,120], these data are of major interest. Although the respective roles of each amino acid used in the mixture cannot be determined in such studies, it is worth noting that the indispensable amino acid threonine is abundant in mucins and the optimal synthesis of mucins requires a relatively high supply of threonine [121]. Supplementation with a mixture of glutamate, methionine, and threonine (0.57, 0.30, and 0.50 g/day, respectively) given in the mucosal healing phase after chemical induction of colitis in rats improves the colonic mucosal regeneration/reepithelialization after supplementation for 10 days [122]. Here again, the respective roles of each of the three amino acids in the mixture could not be delineated, but glutamate has been identified as a major energy substrate in colonocytes and as a precursor for the synthesis of the dispensable amino acids aspartate and alanine, as well as for the synthesis of the tripeptide glutathione [123]. This latter compound is involved in the maintenance of the intracellular redox status and in the control of the intracellular concentration of both the oxygen and nitrogen reactive species [124,125]. These reactive species are produced at abnormal levels in inflammatory bowel diseases [126]. Regarding methionine, this indispensable amino acid is largely metabolized in the intestine and is a precursor of cysteine, another precursor for glutathione synthesis [127]. In a mice model with experimentally induced colitis, an elemental diet enriched with a mixture of amino acids (192 g amino acids per kg food) given for 2 weeks was able to slow down colonic mucus degradation [128].

### 3.4. Amino Acid Supplementation During Weight Loss Programs

In the context of the worldwide increased prevalence of obesity in children, adolescents, and adults [129,130], different strategies such as changes in the lifestyle [131], pharmacological treatments [132], and surgical procedures have been implemented [133,134]. Whatever the strategy used for significant weight loss, the loss of fat mass is paralleled by a significant decrease in lean mass, especially regarding skeletal muscles, thus increasing the risk of sarcopenia [135,136,137,138]. The most efficient preservation of lean mass during weight loss periods requires notably the presence of adequate anabolic stimuli [139].

Regular physical exercise, especially resistance-type exercise training, and higher protein intake than the one recommended for the general adult population are both recommended for obese individuals who undertake weight loss programs. In fact, high protein diets, as introduced above, are more satiating than carbohydrates and fats on a basis of an equal energy content. High protein diets are effective in attenuating the loss of lean mass which parallel weight loss [140,141]. Physical exercise appears also effective for reducing the loss of lean mass in cases of body weight reduction [142,143,144] and thus can act in conjunction with high protein diets.

Regarding supplementation with amino acids, the effects of such supplementation on the maintenance of the lean body have been documented in the dietary context of lower protein intake when associated with physical exercise [145]. In obese adults recruited for a four-week randomized controlled trial, meal replacement containing 6 g proteins and enriched with 16 g of a mixture of indispensable amino acids showed beneficial effects on the skeletal muscle cross-sectional area when compared to isoenergetic standard meal replacement containing 16 g protein but no supplement [146]. However, in this trial, the meal replacement mixtures were not isonitrogenous. Amino acid supplementation has been tested in a randomized controlled study with adult men characterized by severe obesity who undergo a 4-week intervention program. This program associates a low-calorie balanced diet with a physical activity program. In such a situation, supplementation with a mixture of indispensable amino acids together with citrate, succinate, and malate was found to be able to increase muscle mass when compared with the control group receiving no supplements [147]. However, the respective roles of the amino acids and of the tricarboxylic acid intermediates could not be identified in this study. Further work is obviously needed to document the interest of supplements with mixtures of specific amino acids associated with high protein diets and physical exercise for limiting the risk of sarcopenia in overweight and obese individuals with marked weight loss.

### 3.5. Amino Acid Supplementation in Metabolic Syndrome

Metabolic syndrome refers to the co-occurrence of several heterogenous parameters including biochemical anomalies, physiological dysfunctions, and anthropometric characteristics. Although the list of these parameters may differ between scientists, they usually gather criteria such as dyslipidemia (such as hypertriglyceridemia, reduced high-density lipoprotein (HDL) cholesterolemia), hyperglycemia, insulin resistance, endothelial dysfunction, elevated arterial blood pressure, and abdominal obesity [148,149]. Other parameters such as microalbumineria and non-alcoholic fatty liver may be included as parts of parameters which define metabolic syndrome [150,151]. The occurrence of several of these parameters in each individual correlates with increased risks of cardiovascular adverse events and of type 2 diabetes [152,153].

From some clinical trials and experimental studies, there are some indications that supplementation with a mixture of indispensable amino acids or individual amino acids such as arginine, leucine, glycine, histidine, and isoleucine can alleviate one or several criteria usually associated with metabolic syndrome.

In a randomized controlled clinical trial, arginine supplementation (8.3 g/day) added to a hypocaloric diet for 21 days, when combined with exercise training in obese and insulin-resistant type 2 diabetic patients, exerts an additive effect on glucose metabolism and insulin sensitivity when compared to dietary restriction and exercise training alone [154]. In a double blind, randomized controlled study with type 2 diabetic patients, arginine in supplements (9 g/day) given for one month significantly improves peripheral and hepatic insulin sensitivity [155]. Furthermore, in a randomized controlled study, a single dose of arginine (8 g) given to hypertensive patients was able to induce post-exercise lower systolic blood pressure [156]. In a double blind, randomized controlled clinical trial, long-term (18 months) oral arginine supplementation (6.4 g/day) improved insulin sensitivity in volunteers with impaired glucose tolerance [157]. Lastly, intravenous infusion of arginine in obese individuals improved insulin sensitivity and restored the insulin-mediated vasodilatation [158]. Some of the effects of arginine may be partly related to the role of this amino acid as a precursor for nitric oxide (NO) synthesis by nitric oxide synthase activities, but this hypothesis must be confronted in new experiments. The rationale at the basis of these studies is that among the numerous physiological functions of NO, the generation of NO from arginine by the vascular endothelium appears essential for the regulation of blood flow and blood pressure [159,160].

Experiments with animal models reinforce the view that supplementation with arginine can improve some of the anomalies which are associated with metabolic syndrome. Indeed, in rats with experimentally induced metabolic syndrome, arginine supplementation given for 8 weeks further increases the beneficial effects of physical training on hypertension, adipose tissue mass, and hepatic steatosis [161]. Also, in a rodent model with metabolic syndrome, arginine supplementation (0.50% in drinking water) given for 4 weeks alleviates hypertension [162]. In addition, chronic arginine supplementation (5% in diet) given for 8 weeks in a rodent model of diet-induced metabolic syndrome improves glucose tolerance, decreases blood pressure, and decreases abdominal fat pads [163]. In a rodent model of insulin resistance and hypertriglyceridemia, arginine supplementation given for 4 weeks counteracts hypertension and hypertriglyceridemia [164]. Of note, in a model of type 2 diabetes in mice, 12-week treatment with arginine in a supplement (1 mg arginine/kg/day) allows increased mitochondrial respiration and biogenesis in cardiomyocytes isolated from animals when compared to the situation in cardiomyocytes isolated from control animals without the supplement [165].

Regarding BCAAs, much more limited information is available about the interest of supplementation with these amino acids in the context of metabolic syndrome than in the case of arginine. The metabolic concepts related to the possible relevance of BCAA supplementation in this situation are complicated. In a paper published more than 5 decades ago, circulating concentrations of these amino acids were found to be elevated in obese humans [166]. The same situation prevails in genetically obese rodents [167,168,169], but the relationship between BCAA concentrations and parameters defining metabolic syndrome in animal models remains obscure. A unifying hypothesis proposes that obesity-associated insulin resistance affects BCAA metabolism (and then the circulating concentrations of BCAAs) [170], but it remains also possible that increased circulating BCAA concentrations play a role in insulin resistance/glucose tolerance which are associated with metabolic syndrome. In animal models, BCAA supplementation does not worsen insulin resistance or glucose tolerance [171], thus suggesting that increased BCAA concentrations are likely more a consequence than a cause of insulin resistance. A recent paper reporting experiments in the mice model indicates that the increased activity in the skeletal muscles of the branched-chain-ketoacid dehydrogenase (BCKDH, the rate-limiting enzyme for BCAA oxidation), decreases (as expected) the circulating concentrations of BCAAs in fasting state. However, despite such decreased BCAA plasma concentrations, no improvement of insulin sensitivity was recorded in this study [172].

However, it is not possible to totally exclude that the ingestion of massive doses of BCAAs may promote detrimental effects in specific situations. For instance, in a mice model of atherosclerosis, supplementation with BCAAs (BCAAs in drinking water at 3 millimolar concentration) accelerates atherosclerosis progression [173].

BCAAs have been tested in clinical trials and animal models for their effect on different parameters associated with metabolic syndrome. In a double blind, randomized controlled trial, leucine supplementation (3 g/day) given for 8 weeks during energy restriction results in improved fat free mass and lean tissue mass compared to the placebo in adults with metabolic syndrome [174]. In mice, leucine supplementation (6%, which corresponds to the amount of leucine in a high protein diet) given for 20 weeks shows beneficial effects on liver triglyceride content and insulin sensitivity [175]. The other BCAA isoleucine (2.5%), when given as a supplement for 4 weeks to dietary-induced obese mice, partly prevents the accumulation of triglycerides in the liver and lowers the epididymal white adipose tissue mass [176].

Other results have been obtained from a few trials aiming to evaluate the interest of supplementation with other amino acids, namely glycine and histidine, in situations of metabolic syndrome. The effects of glycine supplementation (15 g/day) vs. the placebo given for 3 months have been tested in volunteers with characteristics of metabolic syndrome and this amino acid decreases the systolic blood pressure [177]. Glycine supplementation (1%) improves insulin sensitivity in a rodent model of sucrose-induced insulin resistance [178], and, interestingly, this improvement is paralleled by an increased concentration of glutathione in liver.

In a randomized controlled trial with obese individuals with characteristics of metabolic syndrome, histidine supplementation (4 g/day) when given for 12 weeks improves insulin resistance [179].

Lastly, when phenylalanine is given to rats in drinking water (2 g/L) for 28 weeks, it results in an increased hepatic lipid deposition [180]. Such phenylalanine supplementation represents 55 mg phenylalanine per day. This amount can be compared to the usual consumption of phenylalanine from rats’ regular diet, which is 136 mg phenylalanine per day [47].

### 3.6. Amino Acid Supplementation in Miscellaneous Situations

There are some data from preclinical and clinical studies which suggest that amino acid supplementation could be of value in other different situations, notably in cases of inadequate protein intake. Although this narrative review does not claim to be exhaustive, several typical situations are presented here. For instance, regarding osteoporosis prevention, in a randomized study, increased protein intake (from 58 to 69 g per day together with increased calcium intake) in the elderly receiving an adequate vitamin D supply was associated with significant reduction in all fractures, hip fractures, and falls [181]. Because glutamate has been implicated in osteoblast and osteoclast differentiation [182,183,184], because this amino acid displays a mitogenic effect on osteoblasts [185], and lastly because glutamate prevents decreased bone mineral density in the femur and tibia after systemic administration [186], glutamate supplementation has been tested for its effect on bone quality in situations when such quality is compromised. Monosodium glutamate (from 5 to 20 g/kg diet) given for 6 to 12 weeks partly preserves bone quality in mice under moderate protein restriction [187]. However, relatively high doses of glutamate in supplements were used to observe the significant effects in this latter study, and this can be explained by the fact that virtually all glutamate in a regular diet is metabolized by the gut during absorption [188,189]. Then, it would be of interest to test the effects of glutamate in supplements in randomized controlled clinical trials with older volunteers at risk of osteoporosis. The study of the effects of proline supplementation on bone quality would also be of interest because this amino acid can be hydroxylated in the collagen proteins, giving rise to hydroxyproline [190]. Proline and hydroxyproline are abundant in collagens [191]. The test of this hypothesis requires new experimental studies and clinical trials.

Supplementation with amino acids has been tested in hemodialysis patients. Interestingly, oral administration of a mixture containing the nine indispensable amino acids together with nine dispensable amino acids (5.4 g amino acids) administered on interdialytic days to hemodialysis patients was able to counteract the amino acid loss in these patients [192].

In a randomized controlled clinical trial with compensated cirrhotic patients, supplementation with BCAAs (4.06, 3.52, and 3.26 g/day of leucine, isoleucine, and valine, respectively) for 16 weeks improves liver frailty index and muscle mass [193].

Although an observational study found that substituting dietary low-quality carbohydrates with isocaloric animal protein is significantly associated with a lower prevalence of cognitive decline in older people [194], and although higher protein intake is associated with lower odds of subjective cognitive decline in two recent studies [195,196], little is known on the effects of supplementation with one or several specific amino acids on such processes. One study in mice shows that dietary supplementation with indispensable amino acids together with cysteine and arginine increased the mitochondrial biogenesis and endogenous antioxidant response in the hippocampus, to an extent comparable to those elicited by exercise [197]. In this study, the rationale for the supplementation with indispensable amino acids (and notably the BCAAs) was based on the effects of some indispensable amino acids on the activation of the mammalian target of rapamycin (mTOR). Such activation is related to the increased nitric oxide-mediated mitochondrial biogenesis, while arginine (assumed to be in limiting quantity) was used in these experiments as a precursor of nitric oxide. Although these experimental results wait for confirmation in humans, they suggest the beneficial effects of both exercise and complex mixtures of amino acids on this cerebral structure involved notably in memory and learning [198,199]. This pioneering work asks for further research development to document the possible effects of specific amino acids on the process of age-related cognitive decline.

## 4. Safe Utilization of Amino Acids in Supplements

The upper levels of safe intake (ULSI) for amino acids must refer to the highest level of daily amino acid intake (in the form of proteins and as free amino acids) from all sources that is likely to pose no risk of adverse outcomes in healthy individuals. Such ULSI can be determined in reference to the no-observed-adverse-effect level (NOAEL) values and to the lowest-observed-adverse-effect level (LOAEL) values [49]. The Food and Nutrition Board (FNB) and the Institute of Medicine (IOM) in their 2005 report indicate that the tolerable upper intake levels should preferentially refer to NOAEL values when available [45]. The NOAEL values can be defined as the safety threshold below which no adverse effects have been observed. The NOAEL values for all individual amino acids can be determined from clinical trials. The NOAEL values for a given amino acid can be usefully compared with the level of usual consumption (UC) in diet and supplements [47].

NOAEL and LOAEL values have been determined in many more preclinical studies with animal models (most often rats) than in clinical trials with volunteers. The values obtained in preclinical studies cannot be extrapolated to humans because such extrapolation is hazardous. This is notably because the usual amino consumption from all sources in rats is one order of magnitude higher than the consumption of amino acids in humans when expressed as mg amino acids per kg body weight per day. Then, NOAEL and LOAEL values for amino acids determined in rats are more informative when considered in relation with the animal usual consumption (UC). The NOAEL/UC ratios can provide a rough estimation of the relative tolerance of the individual amino acids in this experimental model [47] as detailed below. The calculation of the NOAEL/UC ratios in humans also bring useful information as presented below.

The duration of the dietary intervention with amino acid supplements in clinical trials is rather variable ranging usually from 3 to 13 weeks, and the parameters used as well as the dietary context, age, and sex of individuals often differ between studies. The NOAEL values are generally based on parameters which include anthropomorphic analysis (body weight, body composition, body mass index), dietary and energy intake, blood and urine biochemical analyses, maximal capacity to oxidize amino acids, circulating concentrations of amino acids and their metabolites, gastrointestinal signs (nausea, diarrhea, etc.), questionnaires on the perceived quality of life, adverse events often classified as mild (which require no treatment), moderate (which require brief pharmacological treatment), and severe (which require hospitalization), and clinical parameters such as blood pressure and renal, cardiovascular, and hepatic functions. Only converging results regarding the different parameters measured after amino acid supplementation, and notably the results of clinical laboratory tests commonly used in health checkups (primary endpoints) and adverse event counts (secondary endpoints), can be utilized for the proposal of NOAEL values.

The NOAEL values for amino acids have been determined in clinical trials for histidine, lysine, methionine, phenylalanine, threonine, tryptophan, arginine, glycine, and serine. Regarding the indispensable amino acid histidine, this amino acid was given as a supplement for 4 weeks to men and women, and different parameters including anthropometric analysis, determination of body composition, sleep patterns, dietary intake, blood biochemistry including measurement of histamine (produced from histidine by decarboxylation), and urine analysis were performed. According to the results obtained, a NOAEL value for histidine in supplements of 8 g/day was proposed [200], a value significantly higher than UC for adults in Western countries (2.0 g/day).

The amino acid leucine was given to adult men as a supplement for 8 h and based on parameters such as the upper capacity to oxidize leucine, as well as glycemia, insulinemia, alanine aminotransferase, and ammonemia, it was found that the acute metabolic capacity of the human body for leucine oxidation is 35–38 g/day [201,202]. In elderly men, the upper limit for leucine oxidation is like the value determined in young men [202]. This value is much higher when compared to the UC (5.8 g/day) for adults in Western countries, but it must be underlined here that this upper value was determined in very short-term trials so that no NOAEL value can be proposed for this amino acid. Indeed, NOAEL values need to be determined in medium- and/or long-term trials based on multiple clinical parameters.

Supplementation with lysine added to an ordinary diet performed for different periods of time (up to 36 months) can cause symptoms related to the gastrointestinal tract such as nausea and diarrhea. These results led to the proposal in a literature review of a provisional NOAEL for lysine in food and supplements equal to 6 g/day [203]. This later value is close to the UC for adults (5.1 g/day), thus suggesting that relatively small amounts of lysine in supplements may lead to mild adverse events. Then, based on these data, supplementation with lysine in countries where lysine UC is close to the NOAEL value (such as the situation in Western countries) for this amino acid should be avoided except in subpopulations where lysine uptake from food has been robustly documented as clearly insufficient compared to the requirement.

When methionine is given as a supplement for 4 weeks to older adults (men and women), based on plasma homocysteine concentration used as the primary determinant (homocysteine is produced from methionine in three steps), but also on numerous other biochemical blood variables, the NOAEL value for methionine in supplements was found to be equal to 3.2 g/day [204]. In fact, plasma homocysteine was elevated with the highest dose of methionine in supplements (that is, 91 mg/kg body weight per day, and thus 6.4 g methionine per day for an individual weighing 70 kg). The NOAEL value for methionine is above the UC for adults (1.7 g/day).

Regarding phenylalanine, when given as supplement to male volunteers for 4 weeks, based on parameters including blood biochemistry and the absence of treatment-related adverse events, the NOAEL value for this amino acid in supplements was found to be 12 g/day [205], a value which can be compared to the UC of this amino acid for adults (3.3 g/day). The difference between the NOAEL value and UC suggests that phenylalanine in supplements is well tolerated in humans.

The NOAEL value was determined for threonine in supplements in a double blind randomized controlled trial performed for 4 weeks in men. Based on anthropometric parameters, blood chemistry (including amino acid concentrations), dietary intake, and the detection of adverse events, a NOAEL value of 12 g/day was proposed [206]. Interestingly, when measured after an overnight fast in plasma obtained from volunteers at the end of the 4-week trial, a marked specific increase in threonine concentration was measured after consumption of high doses of threonine in supplements (between 6 and 12 g/day) when compared to the placebo value. Since i. the amino acid plasma concentrations can be considered in the first place as the net result of intestinal absorption and uptake/release from tissues. and since ii. the absorption of amino acids resulting from dietary protein digestion is minimal after an overnight fast [207,208], these results suggest possible modifications of threonine metabolism after 4-week supplementation with this amino acid. These modifications of threonine circulating concentrations were not associated with any adverse effects in the conditions of the clinical study. Further studies are required to identify the potential metabolic steps modified by threonine medium-term intake (e.g., saturation of threonine catabolic pathways, impaired threonine tissue-specific uptake or release). In addition, it would be of interest to determine if the modification of threonine plasma concentration after a 4-week supplementation with this amino acid is retained in the long-term period, and if it may have functional consequences. When compared with the UC for threonine in adults (2.9 g/day), the NOAEL value is about four times higher.

For tryptophan, the NOAEL value for supplementation was determined in a double blind randomized controlled clinical study performed with female volunteers using values from blood and urine biochemistry (notably the tryptophan metabolites nicotinamide, kynurenin, and kynurenic acid in urine) and the profile of mood states category measurement. Of note, the urinary excretion of nicotinamide and of the metabolic intermediates formed in the tryptophan catabolic pathways (such as kynurenine, kynurenic acid, 3-hydroxykynurenin, 3-hydroxyanthranilic acid, and quinolinic acid) was increased in proportion to the tryptophan doses in supplements, suggesting a dose-dependent physiological catabolism of tryptophan by participants [209]. Incidentally, the authors of this study proposed the measurement of urinary excretion of the intermediates formed between tryptophan and quinolinic acid as indicators of tryptophan ingestion. From this study, the tryptophan NOAEL value was proposed to be 5 g/day. This value, when compared to the UC in adults (0.8 g/day), suggests that tryptophan is well tolerated when given in supplements.

Regarding the dispensable amino acids arginine and serine, NOAEL values have been determined in volunteers. Regarding arginine, in a randomized controlled clinical trial with overweight or obese but otherwise healthy volunteers (men and women), the NOAEL value was determined from blood biochemistry measurement, as well as measurements of cardiovascular, renal, and hepatic parameters. The results obtained indicate that supplementation with arginine at a dose of 30 g/day for three months can be considered as safe [210]. Interestingly, in this study, supplementation with 30 g arginine per day reduced systolic blood pressure in women. In their review of the literature, Shao and Hathcock, after considering the observed safe level (OSL) of arginine in supplements, propose to not go beyond 20 g arginine per day [211]. OSL values, which differ from NOAEL values, can be defined (usually in medical and pharmaceutical sciences) as the doses of a given compound for which no adverse effects are observed. By reviewing the randomized controlled trials performed with arginine supplementation in volunteers, Kuramochi and collaborators recently proposed a lower NOAEL value (7.5 g/day) for this amino acid in supplements because of the light gastrointestinal symptoms recorded in some studied for some participants at higher doses [212]. From the available results obtained in published peer-reviewed clinical studies, it is recommended for safety reasons to use the lowest NOAEL value proposed. However, more generally, it is advisable to compare in some specific situations the beneficial effects of supplementation with amino acid(s) to the possible light adverse effects in a classical perspective of risk–benefit ratio evaluation. Then, if we consider the lower value of NOAEL proposed for arginine (thus 7.5 g/day) and if we compare this value with the UC (4.2 g/day), this amino acid appears to be relatively well tolerated since the NOAEL/UC ratio is equal to 1.8.

For serine, the NOAEL value has been determined in male volunteers based on the circulating concentrations of biochemical analytes (including amino acids) and found to be 12.0 g/day in supplements [205]. When compared with the UC for serine (3.9 g/day), the NOAEL value for this amino acid is approximately three times higher.

Determination of NOAEL values has also been made for the non-proteinogenic amino acids ornithine and citrulline which are used as dietary supplements in different situations [213,214,215]. The NOAEL values for ornithine and citrulline in supplements were determined in a 4-week clinical trial performed in men based on the measurement of circulating biochemical analytes (including amino acid) concentrations, the occurrence of adverse events, and mental self-assessment [216]. According to this study, the NOAEL values for ornithine and citrulline were 12 g/day and 24 g/day, respectively. The literature review performed on clinical studies dealing with the oral intake of ornithine up to 22 weeks of consumption has recently confirmed a NOAEL value equal to 12 g/day for this amino acid in supplements [217]. The NOAEL values for the amino acids for which it has been determined are summarized in Table 3. Importantly, the NOAEL values presented have been generally determined in healthy young adults, and efforts remain to be made to determine the NOAEL values in older individuals and in volunteers with prepathological/pathophysiological situations where amino acid supplementation appears beneficial.

For the other indispensable amino acids (isoleucine and valine), and dispensable amino acids (alanine, asparagine, aspartate, cysteine, glutamate, glutamine, glycine, proline, and tyrosine), no NOAEL or LOAEL values have been determined in clinical trials. For glutamine, an observed safe level of 14 g/day has been proposed for human consumption [210]. Then, only data from animal studies are available. Although, as previously said, it is by no way possible to extrapolate from animal models to humans, some studies in rats can bring useful information [47]. These rat studies suggest that the NOAEL values can be much different for some amino acids according to the sex of animals, raising the possibility of differential tolerance to supplements between males and females in other mammals including humans. Indeed, the NOAEL values for valine and tyrosine are higher in male than female rats [218,219], while for tryptophan, the NOAEL value is higher in female than male rats [220].

By using the NOAEL and UC values determined in rats, it is possible to calculate the NOAEL/UC ratios for some amino acids. Such calculations give a rough estimation of the tolerance of amino acids in supplements for rats [47]. In this process, alanine appears well tolerated in rats (NOAEL/UC ratio is 6.9), followed by serine (4.8), leucine (3.7), isoleucine (3.1), proline (2.3), and aspartate (1.0), with this latter amino acid thus appearing to be the least tolerated amino acid among this list. Indeed, after 90 days of supplementation, toxic effects of aspartate (2.5% and 5.0%) on the kidneys and salivary glands are recorded [221], leading to proposed NOAEL values of 697 and 715 mg/kg body weight per day for male and female rats, respectively. These values are not much different when compared to the rat UC when animals are fed with regular purified diets [222]. The mechanisms underlying aspartate toxicity remain to be identified. Whatever they are, these preclinical values impose precautions for future clinical trials with aspartate supplementation in volunteers.

## 5. Conclusions and Prospects

The NOAEL values for amino acids in supplements in humans have been determined for only nine amino acids among the twenty common amino acids found in proteins. In addition to these nine proteinogenic amino acids, NOAEL values have been ascertained for the non-proteinogenic amino acids ornithine and citrulline. Thus, for the other eleven proteinogenic amino acids, additional clinical trials are needed to estimate the upper levels of safe intake of these amino acids in supplements.

What could be the mechanistic reasons, beyond the clinical signs, that would explain the deleterious effects of excessive amounts of amino acids in supplements? Although we still have a limited vision of the mechanisms involved, some studies suggest that excessive amounts of amino acids could disturb the normal overall metabolism of these compounds. For instance, it is well known that the amino acid transporters situated in the luminal brush border and in the baso-lateral membranes of the enterocytes can transport several amino acids with different affinities [223]. Furthermore, a particular amino acid may modulate the transport and metabolism of other amino acids along the small intestine [224]. Then, a supply of an excessive amount of one given amino acid may have an impact on the transport of other amino acids and may then affect their metabolism firstly in enterocytes and then in peripheral tissues after absorption [225]. In fact, competition for shared amino acid transport systems has been described for several decades in different organs and cells including the small intestine [226], brain [227], kidney [228], and macrophages [229]. Excessive concentration and then intake of a single amino acid may saturate the corresponding amino acid transporter, thus hindering the uptake of other amino acids. For instance, the kinetic study of alanine transport by the apical surface of human enterocytes in the presence of glutamine indicates competition for the transport of these two amino acids within enterocytes [230]. In another study with human intestinal epithelial cells, kinetic analysis with glutamine used at increasing concentrations in the presence of lysine indicates competition for the transport of these two amino acids across the brush border membranes [231]. Using brush border membranes obtained from pig jejunal enterocytes, the kinetic study of the inhibition of glutamine uptake by leucine or cysteine points to the inhibition of the transport of glutamine by the two other amino acids [232].

In addition, the kinetics of amino acid absorption for amino acids in free form in supplements on the one hand, and for amino acids originating from proteins on the other hand, represent potentially important parameters for the metabolic and physiological consequences of amino acid supplementation. Such modifications may affect amino acid-dependent physiological processes.

From this point of view, the capacity for metabolic adaptation after medium-term amino acid supplementation should be considered to evaluate the possible impact of such supplementation in humans. To give a typical example of a metabolic adaptation observed in animal models, the ingestion for 7 days of a high protein diet (58% casein in weight as protein source) was found to increase ammonia (taken as the sum of NH_4_^+^ and NH_3_) concentrations in both the luminal colonic fluid and in the portal vein when compared to the situation observed in rats receiving a control isocaloric diet containing 20% casein [233]. Although the ammonia concentration in the colon remains higher during the 7 days of experiments after a high protein diet (when compared with the control diet), the ammonia concentration in the portal vein was found to be elevated for 4 days and then goes back to the basal value recorded in the control experiments. These modifications in the ammonia concentrations in animals fed with a high protein diet were paralleled by marked increases in arginase activity and the production of ornithine from arginine within the colonic epithelial cells during the 7 days of experiments. Such an increased capacity of colonocytes to synthesize ornithine likely corresponds to an elevated requirement of ornithine for the elimination of higher concentrations of ammonia in the blood in the liver urea cycle [234]. These results suggest that colonocytes participate in the increased capacity of the liver urea cycle after high protein diet consumption.

Considering the capacities of metabolic adaptation in tissues is critical to distinguish between the acute and potential chronic effects of the modifications of the dietary conditions both in terms of protein intake and of amino acid supplementation. Up to now, only limited information is available regarding the metabolic adaptation capacities of the human tissues and organs in response to modified amino acid intake.

In specific situations of insufficient protein intake, as presented in this narrative review, supplementation with individual amino acids with relevant quantities may be recommended to allow optimal physiological and metabolic functions. This is the case for instance for subpopulations among the elderly with protein–energy deficiency status. According to clinical trials performed in elderly populations with sarcopenia (or at risk for sarcopenia), such amino acid mixtures should contain a relatively high content in leucine among the other amino acids. Such dietary supplementation appears more effective when combined with regular physical exercise.

In athletes, branched-chain amino acids appear able to increase muscle protein synthesis during the recovery phase. In experiments with healthy volunteers receiving leucine by intravenous infusion, the results suggest that this BCAA decreases the overall protein degradation in the body. A possible supplementation with BCAAs in athletes should be reasoned in relation to the recommended intake of proteins in this subpopulation. As indicated above, the recommended protein intake in athletes is 1.6 to 2.2 times higher than in the general adult population.

More generally, the question of the supply of amino acids above the usual requirements for obtaining optimized metabolic and physiological outcomes in some specific situations remains to be answered in an unequivocal manner in each situation. For instance, as presented above, after an episode of experimental intestinal inflammatory flare in rodents, the mucosal healing phase seems to require a higher uptake of alimentary proteins than in healthy animals, and some specific amino acids in supplements appear efficient for such a process. These results encourage further innovative but safe experimental and clinical studies on the protein and specific amino acid requirements during the healing phase after inflammatory episodes.

Importantly, the multifunction roles of amino acids both as precursors for the synthesis of macromolecules (proteins and the purine and pyrimidine rings in RNAs and DNA) and as precursors for the synthesis of bioactive compounds should be considered when evaluating the relevance of supplementation with amino acid(s) in each situation. In fact, these numerous amino acid-derived compounds exert central metabolic and physiological roles, and the chemical identity and roles of these compounds are different according to the amino acid considered [1]. In addition, specific amino acids are precursors for ATP synthesis and are involved as energy substrates in numerous cell phenotypes.

During weight loss programs in obese volunteers, only few data are available regarding the relevance of supplementation with mixtures of specific amino acids for the maintenance of the lean mass. In clinical trials with volunteers characterized by biological parameters associated with metabolic syndrome, the indispensable amino acids leucine and histidine, as well as the conditionally indispensable amino acids arginine and glycine, have shown some beneficial effects on the underlying criteria which define this syndrome.

**Figure 3:**
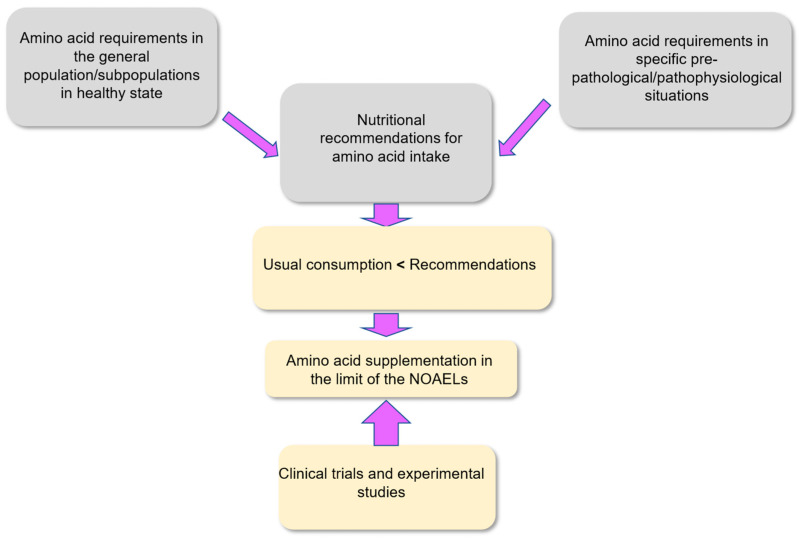
Paradigm for amino acid supplementation. The amino acid requirements in different physiological, pre-pathological, and pathophysiological situations are used to determine the nutritional recommendations for the intake of the different amino acids. When the usual consumption of each amino acid is lower than the recommendation, amino acid supplementation can be recommended for optimal metabolic and physiological functions in the limits of the no-observed-adverse-effects levels (NOAELs). The doses of amino acids in supplements, as well as the NOAEL values, are determined in clinical trials. Usual consumption of amino acids is determined from intake from dietary proteins and from intake of dietary amino acids in their free form.

Overall, the challenge for the coming years is to identify more precisely the different physiological and pathophysiological situations, in different subpopulations, for which supplementation with amino acids will prove to be useful from a preventive or curative perspective (Figure 3).

In future studies, the long-term effects of supplementation with amino acid(s) should be considered as well as the dose–response relationships between the doses of amino acid(s) used in supplements and the effects observed. In addition, future research aiming at determining the recommendations for amino acid supplementation should consider the targeted specific subpopulations including the vulnerable groups (like for instance the sarcopenic elderly and the IBD patients in remission). In this regard, the specific requirements for proteins and specific amino acids need to be determined in each targeted subgroup of the population.

Although for safety reasons clinical trials with healthy adults are recommended as first steps of exploration, further trials with volunteers in different prepathological/pathophysiological situations, of different ages, and of both sexes are necessary. Clearly, supplementation with amino acid(s) requires additional precautions in individuals with pre-existing metabolic and physiological dysfunctions which may result in the lowering of the NOAEL values when compared with values determined in healthy subjects. Medical supervision is advisable before amino acid supplementation in such situations. To give a concrete example of such a clinical situation, as introduced above, in the sarcopenic elderly with chronic kidney disease and loss of appetite, the choice for the recommendation of the optimal protein intake is tricky. From this point of view, supplementation with a combination of specific amino acids represents a valuable and relevant alternative to a simple increase in alimentary protein consumption.

**Figure 4:**
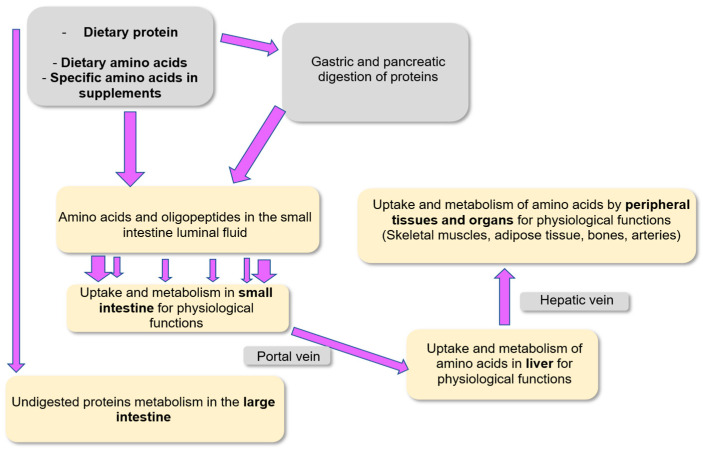
Schematic representation of the fate of amino acids originating from diet and supplements in the human body. Specific amino acids in supplements together with amino acids originating from gastric and pancreatic digestion of dietary proteins are taken up and metabolized by absorptive epithelial cells of the small intestine for physiological functions. The large proportion of unmetabolized amino acids is released in the portal vein and reaches the liver cells and then the peripheral tissues and organs.

The identification of the doses of amino acids, either in individual or mixture form, together with the determination of the usage durations which are efficient on target organs and tissues and safe, is needed. Figure 4 summarizes schematically the fate of amino acids originating from diet and supplements in the body. Amino acids, whatever their origin (diet or supplement), are absorbed after meals by transporters present in the apical and basolateral membranes of enterocytes. The transport of amino acids within these cells can be competitive depending on the relative luminal concentrations of the different amino acids. During their transfer from the small intestine luminal fluid to the venous capillaries, a minor portion of amino acids is used by enterocytes for metabolic and physiological functions. The bulk of amino acids (thus unmetabolized amino acids) moves to the liver cells where they are imported and partly metabolized allowing different physiological functions. Amino acids in the general circulation are imported by peripheral tissues and organs and here again used for physiological functions. The intestine, liver, and peripheral tissues and organs such as skeletal muscles, adipose tissue, bone, and arteries have been targeted by amino acid supplementation in specific situations. Incidentally, a minor proportion of undigested proteins can reach the large intestine where they are degraded by bacteria which use them for their own metabolism and produce numerous metabolites. These metabolites have been shown to be active both on the host’s tissues and organs [113] and on the microbial activity [235]. Limited information is available regarding the impact of amino acid supplementation on microbiota metabolism and physiology, and on the consequences for the human host.

Finally, regarding the safety of amino acids in supplements, the NOAEL values for all the eleven amino acids for which we have no values (even in healthy volunteers) need to be determined in future clinical trials to obtain robust reference values for users. In that regard, the choice of the relevant metabolic and physiological parameters in each situation is of major importance.

## Figures and Tables

**Table 1:** Average protein requirements along the life cycle in healthy subpopulations.

Subpopulations	Average Protein Requirements (g per kg Body Weight per Day)
Infants (0–3 years)	0.76–1.80
Children (4–10 years)	0.70
Adolescents (11–18 years)	0.63–0.72
Adults	0.65
Pregnant women	0.65–0.73
Breast-feeding women	0.80
Elderly	1.0–1.6

Note: The values for infants, children, adolescents, adults, pregnant women, and breast-feeding women are from references [26,36], while for the elderly, the value is from the references [38,39,40,41].

**Table 2:** Requirement of indispensable amino acids in adults and usual consumption in North America and Europe.

Indispensable AA	Requirements			Usual Consumption	
	(mg/kg BW Per Day)				
	FNB/IOM ^1^	WHO ^2^	AFSSA ^3^	FNB/IOM ^4^	NMCD ^5^
Histidine	11	10	11	31	28
Isoleucine	15	20	18	51	45
Leucine	34	39	39	87	80
Lysine	31	30	30	75	71
Methionine	10 ^a^	10	15 ^b^	25	23
Phenylalanine	27 ^c^	25 ^c^	27 ^d^	49	46
Threonine	16	15	16	43	40
Tryptophan	4	4	4	13	11
Valine	19	26	18	57	54

Note. Requirements and usual consumption are given in mg amino acid per kg body weight per day. ^1^ Values from the Food and Nutrition Board/Institute of Medicine (FNB/IOM) 2005 report [45]; ^2^ Values from the World Health Organization (WHO) 2007 report [46]; ^3^ Values from the French Agency for Food Safety (AFSSA) 2007 report [36]; ^4^ Usual consumption (UC) from food and supplements in North America for all life stage groups; ^5^ UC values for European adults from Iguacel et al. Nutr. Metab. Cardiovasc. Dis. (NMCD) 2022 [48]. ^a^ Methionine requirement in the presence of an excess of dietary cysteine; ^b^ Sulfur-containing amino acids; ^c^ Phenylalanine and tyrosine, ^d^ Aromatic amino acids. AAs are amino acids and BW is body weight.

**Table 3:** No-observed-adverse-effect-level (NOAEL) values for amino acids in supplements in humans.

Amino Acids	NOAEL Values (g/day)	References
Histidine	8.0	[200]
Lysine	6.0	[203]
Methionine	3.2	[204]
Phenylalanine	12.0	[205]
Threonine	12.0	[206]
Tryptophan	5.0	[209]
Arginine	7.5	[212]
Serine	12.0	[205]
Ornithine	12.0	[216]
Citrulline	24.0	[216]

Note: The available NOAEL values for amino acids in supplements for humans are given for the eight proteinogenic amino acids and for the two non-proteinogenic amino acids (ornithine and citrulline).

## Data Availability

No new data were created or analyzed in this review. Data sharing is not applicable to this article.
